# Artificial Intelligence for the Identification of Vascular Imaging Biomarkers in COPD: Redefining Phenotypes and Enabling Precision Care

**DOI:** 10.3390/jcm14207134

**Published:** 2025-10-10

**Authors:** Gaetano Rea, Pasquale Ambrosino, Claudio Candia, Mauro Maniscalco

**Affiliations:** 1 Department of Radiology, Azienda Ospedaliera dei Colli, Monaldi Hospital, 80131 Naples, Italy; gaetano.rea71@gmail.com; 2 Istituti Clinici Scientifici Maugeri IRCCS, Scientific Directorate of Telese Terme Institute, 82037 Telese Terme, Italy; 3 Department of Biomedicine, Neurosciences and Advanced Diagnostics, University of Palermo, 90127 Palermo, Italy; c.candia@outlook.it; 4 Istituti Clinici Scientifici Maugeri IRCCS, Pulmonary Rehabilitation Unit of Telese Terme Institute, 82037 Telese Terme, Italy; 5 Department of Clinical Medicine and Surgery, Federico II University, 80131 Naples, Italy

Chronic obstructive pulmonary disease (COPD) constitutes a persistent and substantial global health challenge, characterized by pronounced clinical and biological heterogeneity [1], often resulting in significant functional impairment, long-term disability, and increased rehabilitation needs [2,3]. So far, although pulmonary function testing remains the cornerstone for diagnosing COPD and for stratifying airflow limitation severity [4], phenotyping has predominantly relied on clinical variables, such as dyspnea severity and exacerbation history [4]. Objective tools, including laboratory testing and conventional imaging, have also been employed to evaluate peripheral eosinophilia and to characterize parenchymal abnormalities, such as emphysema and bronchial wall thickening [5]. Together, these parameters have shaped the clinical classification and therapeutic strategies adopted in the last fourteen years. Emerging evidence now highlights the critical yet often underappreciated role of pulmonary vascular remodeling in disease progression and patient outcomes [6]. In this scenario, recent advances in artificial intelligence (AI) and deep learning (DL) have revolutionized thoracic imaging analysis, enabling the extraction of precise quantitative vascular biomarkers from routine and high-resolution chest computed tomography (CT) scans [7]. Therefore, the integration of AI-derived vascular metrics may unveil novel and critical endotypic dimensions, offering the potential to redefine disease stratification frameworks, enhance prognostic accuracy, and advance COPD care towards a fully individualized approach [8].

As a matter of fact, several respiratory conditions, including COPD, are increasingly recognized as systemic disorders, with pulmonary vascular involvement representing a critical interface between systemic dysregulation and localized pulmonary pathology [9]. The pulmonary vasculature, serving as the interface between the circulatory system and the respiratory parenchyma, is particularly susceptible to systemic inflammatory, oxidative, and hemodynamic insults [10]. Subtle disturbances in the pulmonary vasculature, including vessel pruning, microvascular rarefaction, endothelial dysfunction, and altered perfusion distribution, thus contribute to functional impairment, heightened risk of exacerbations, and progressive right ventricular strain, ultimately leading to the onset of pulmonary hypertension in COPD [11,12]. Interestingly, these vascular changes may arise independently of (or even precede) emphysematous destruction and airway remodeling, often eluding detection by conventional imaging or functional tests [13]. Quantitative assessment of complex vascular alterations offers critical insight into the vascular endotype of COPD, with metrics such as total blood volume, small pulmonary vessel volumes, and their derived ratios providing objective measures of pulmonary vascular health [14]. These parameters elucidate underlying pathophysiological mechanisms, allowing clinicians to delineate patient subgroups whose disease trajectories are predominantly driven by vascular pathology, with major implications for identifying pulmonary hypertension in advanced COPD [15].

The application of AI, particularly sophisticated DL algorithms, has transformed the analytic potential of chest CT imaging [16]. Advanced computational platforms, both commercial and open-source, now enable fully automated, highly reproducible segmentation and quantification of pulmonary vasculature from standard CT or high-resolution CT (HRCT) scans. This facilitates robust extraction of intricate vascular metrics across large datasets and routine clinical workflows [17].

The distinct advantages of AI-based analysis lie in its ability to detect sub-visual biomarkers, identifying minute vascular alterations that are imperceptible to the human eye and may serve as early indicators of disease progression or subtle treatment effects [17]. Moreover, AI enhances diagnostic accuracy by providing objective and quantitative data that complement visual assessments, thereby improving diagnostic consistency and precision across a wide range of clinical scenarios and imaging contexts [18]. Finally, the scalability of AI enables high-throughput analysis across large patient cohorts, accelerating the discovery of novel imaging biomarkers and their associations with clinical outcomes, functional decline, and therapeutic responses [17]. This capability may not only facilitate broader population-level investigations but also support more rapid real-world implementation by enabling the automated processing of imaging data in routine workflows.

The incorporation of AI-derived vascular biomarkers into COPD assessment holds transformative potential across multiple domains. First, it may allow for more refined phenotyping and endotyping by identifying distinct vascular patterns that go beyond traditional spirometry or parenchymal classifications. This approach offers a deeper understanding of individual disease profiles and enables the identification of patients with predominant vascular pathology [16]. In addition, quantitative vascular metrics have been consistently associated with an increased risk of acute exacerbations, pulmonary hypertension, and cardiovascular complications [15]. Thus, their integration into prognostic models may enhance risk stratification and support earlier, more proactive interventions [16,17]. Furthermore, recognition of a patient’s specific vascular signature may directly inform therapeutic decision-making. For instance, identifying a prominent vascular component could support the use of treatments aimed at modulating pulmonary vascular tone or addressing cardiac comorbidities, thus promoting a truly personalized approach to care [19]. Lastly, longitudinal monitoring of vascular biomarkers may offer an objective means of tracking disease progression and therapeutic response, complementing traditional spirometry and clinical endpoints and enabling a more dynamic adaptation of treatment strategies over time [20].

Despite these significant scientific advances, several critical challenges must be addressed before AI-derived vascular biomarkers can be widely adopted in clinical practice [8]. First, standardization is essential. The development of universally accepted definitions for vascular imaging metrics, along with robust and reproducible measurement protocols, is necessary to ensure consistency and comparability across studies and healthcare settings [21]. Equally important is the need for rigorous validation. AI algorithms must undergo multicenter evaluation in diverse patient populations to confirm their generalizability, reliability, and clinical relevance. Without this step, the risk of overfitting and limited applicability may undermine their translational value [8]. Clinical integration also remains a major hurdle. To ensure that advanced imaging analytics contribute meaningfully to care, they must be seamlessly embedded into existing clinical workflows and decision support systems. This will help clinicians access and interpret these data in a timely and actionable manner [22]. Finally, the successful implementation of these tools will depend on sustained interdisciplinary collaboration. Effective translation from research to practice requires in fact strong partnerships among pulmonologists, radiologists, data scientists, and industry stakeholders. Only through such coordinated efforts can the promise of AI-driven vascular phenotyping be fully realized at the bedside [23].

Overall, the emergence of AI-driven vascular biomarker extraction from chest CT imaging represents a fundamental paradigm shift in the phenotyping and management of COPD. By illuminating the previously underexplored vascular dimension of this complex disease, these technologies hold the potential to refine diagnostic algorithms, enhance risk stratification, and inaugurate a new era of precision medicine. As evidence continues to accumulate, integrating these sophisticated vascular metrics into clinical guidelines may become central to individualized COPD care, not only in terms of pharmacologic strategies but also in tailoring comprehensive rehabilitation programs to specific vascular phenotypes [24]. This integration offers renewed therapeutic avenues and clearer prognostic insights for millions of patients worldwide.
